# Synergistic Effect of Subtoxic-dose Cisplatin and TRAIL to Mediate Apoptosis by Down-regulating Decoy Receptor 2 and Up-regulating Caspase-8, Caspase-9 and Bax Expression on NCI-H460 and A549 Cells

**Published:** 2013-05

**Authors:** Xiaoyan Zhang, Jing Zhao, Wenyan Zhu, Hongfeng Gou, Dan Cao, Yu Yang, Ying Huang, Cheng Yi

**Affiliations:** 1 Department of Medical Oncology, Cancer Center of West China Hospital, Sichuan University, Chengdu, Sichuan Province, China; 2 Department of Pathophysiology, West China School of Preclinical and Forensic Medicine, Sichuan University, Chengdu, Sichuan Province, China; 3 First Department of Oncology, Hebei General Hospital, Shijiazhuang, Hebei Province, China

**Keywords:** Apoptosis, Cisplatin, DcR2, Nonsmall cell lung cancer, Subtoxic-dose, TRAIL

## Abstract

***Objective(s)*** *:* Although tumor necrosis factor-related apoptosis-inducing ligand (TRAIL) can selectively induce apoptosis in tumor cells, more than half of tumors including non-small cell lung cancer (NSCLC) exhibit TRAIL-resistance. The purpose of this study was to determine whether subtoxic-dose cisplatin and TRAIL could synergistically enhance apoptosis on NSCLC cells and investigate its underlying mechanisms**.**

***Materials and Methods:*** NCI-H460 and A549 cells were treated with TRAIL alone, cisplatin alone or combination treatment in this study. The cytotoxicity was evaluated according to Sulforhodamine B assay, and apoptosis was examined using Hoechst 33342 staining and flow cytometry. The mRNA and protein levels of TRAIL receptors and apoptotic proteins including caspase-8, caspase-9, Bcl-2 and Bax were determined by RT-PCR and Western blotting, respectively.

***Results:*** Our results showed that NCI-H460 cells were sensitive to TRAIL, whereas A549 cells were resistant. However, subtoxic-dose cisplatin could enhance the both cells to TRAIL-mediated cell proliferation inhibition and apoptosis. The underlying mechanisms might be associated with the down-regulation of DcR2 and up-regulation of Caspase-8, Caspase-9 and Bax.

***Conclusion:*** Subtoxic-dose cisplatin could enhance both TRAIL- sensitive and TRAIL- resistant NSCLC cells to TRAIL-mediated apoptosis. These findings motivated further studies to evaluate such a combinatory therapeutic strategy against NSCLC in the animal models.

## Introduction

Lung cancer is the leading cause of cancer death, and non-small cell lung cancer (NSCLC) is its most common form, accounting for 80-85% of all lung cancers (1). Surgery, chemotherapy and radiotherapy are considered to be routine treatments for NSCLC. Approximately 75% of these patients have to choose chemotherapy due to advanced or metastatic cancer (2). Unfortunately, chemotherapy often shows poor response rates for NSCLC due to drug resistance. Therefore, new approaches designed to reactivate cell death or apoptosis programs are urgently desirable. 

Tumor necrosis factor-related apoptosis-inducing ligand (TRAIL), a member of the tumor necrosis factor (TNF) superfamilies, is a candidate for cancer therapy due to its ability to selectively trigger apoptosis in tumor cells but not normal cells (3-5). Thus, TRAIL is a promising and novel NSCLC therapeutic agent. TRAIL-induced apoptosis can be modulated by many factors. It is well known that there are five TRAIL-receptors including death receptor-4 (DR4), death receptor-5 (DR5), decoy receptor 1 (DcR1), decoy receptor 2 (DcR2), and osteoprotegerin (OPG) (6). DR4 and DR5 are involved in activation of extrinsic apoptosis pathway, whereas DcR1, DcR2 and OPG regulate negatively apoptosis (7). 

Ligation to DR4/DR5 leads to the formation of a death-inducing signaling complex (DISC) which activates pro-caspase 8. Activated caspase 8 directly activates the executioner caspases, and cleaves the BH3-only protein Bid, leading to the formation of Bax/Bak megachannels in the outer mitochondrial membrane (8). Then cytochrome C is released into cytosol to trigger activation of procaspase 9 and the executioner caspases (9). Bcl-2 can block TRAIL-induced apoptosis in type II cells. The intracellular ratio of Bax to Bcl-2 is thought to be critical in determining cellular response to apoptotic stimuli (10). DcR1 and DcR2 are unable to induce directly apoptosis. However, their overexpression can block TRAIL-mediated apoptosis in some cells (7), and then results in TRAIL-resistance. Recent studies have shown that down-regulation of DcR2 might be involved in sensitizing TRAIL-induced cell death or apoptosis (11, 12). 

Although TRAIL has exhibited potent activities, some tumor cells including prostate, colon, and glioma still show resistant to it (11-14). TRAIL-resistance is likely to be mediated by multiple defects in signaling pathway including inactivating mutations in DR4 and DR5, loss of the initiator caspase-8 and Bax, and overexpression of Bcl-2 (13, 15-17). The overexpression of DcR1 and DcR2 is also involved in TRAIL-resistance. Therefore, some tumor cells need sensitizing agents to promote TRAIL-induced apoptosis. Recent studies have reported that some chemotherapy agents could sensitize tumor cells to TRAIL-mediated apoptosis (18-20).

Cisplatin has been recognized as one of the first-line chemotherapeutic agents for NSCLC (21, 22). However, routine chemotherapy schemes usually induce considerable toxicities (23-25). In this study, we investigated the effects of subtoxic-dose cisplatin combined with TRAIL on NSCLC cells. The combination treatment could lighten toxicities of cisplatin and enhance NSCLC cells to TRAIL-mediated apoptosis. Previous studies had shown that a combination treatment of TRAIL and conventional dose cisplatin (>7.5 μg/ml) was effective in some tumor cells (26, 27), but few studies on TRAIL combined with subtoxic-dose cisplatin were reported in NSCLC cells, and its underling mechanism was also not well understood. In this study, we aimed to determine whether subtoxic-dose cisplatin could enhance both TRAIL-resistant and TRAIL-sensitive NSCLC cells to TRAIL-mediated apoptosis, and explore the possible mechanisms.

## Material and Methods

***Materials and antibodies***

TRAIL was provided by DiAo Pharmaceutical Group Co., Ltd (Chengdu, China). Cisplatin was supplied by Yunnan Gejiu Bio-pharm. Co., Ltd (Yunnan, China). Sulforhodamine B (SRB) was obtained from Sigma (St Louis, MO, USA). RPMI 1640 medium and trypsin were purchased from Gibco (Grand Island, NY, USA). Both the Hoechst staining kit and annexin V-FITC & PI kit were purchased from Jingmei Biotech Co., Ltd (Shanghai, China). One-step RT-PCR kit was obtained from Takara Biotechnology Co., Ltd (Dalian, China). Polyvinylidene difluoride (PVDF) membrane was purchased from Millipore (Billerica, MA, USA). Mouse monoclonal antibodies for Bcl-2, Bax and β-actin and rabbit polyclonal antibody for Caspase-8 p20 were purchased from Santa Cruz Biotechnology (Santa Cruz, CA, USA). Rabbit polyclonal antibody for Caspase-9 was purchased from Lab Vision (Freemont, CA, USA). Other antibodies including anti-DcR1, anti-DcR2, anti-DR4 and anti-DR5 were obtained from Cell Signaling Technology, Inc (Danvers, MA, USA). Goat antimouse IgG-AP and goat antirabbit IgG-AP were purchased from KPL (Gainthersburg, MD, USA). 

***Cell Lines and cell culture***

Human NSCLC cell lines including NCI-H460 and A549 were respectively provided by DiAo Pharmaceutical Group Co., Ltd (Chengdu, China) and Tumor Immunology Laboratory of West China Hospital (Chengdu, China). They were cultured in RPMI 1640 medium supplemented with 10% (v/v) heat-inactivated fetal bovine serum (Gibco BRL, Carlsbad, CA, USA), 100 U/ml penicillin and 100 U/ml streptomycin. The cultures were incubated at 37°C in a humidified incubator containing 5% CO2.

***Cell proliferation assay***

*In vitro* cell viability was measured using SRB method (28). Briefly, exponentially growing cells were seeded into a 96-well plate (1×10^4^/well) and adhered to the plate for 24 h. Then cells were respectively exposed to serial concentrations of TRAIL alone, cisplatin alone or combination treatment for 48h. After incubation, cells were fixed *in situ* by adding 50 µl ice-cold 50% (w/v) trichloroacetic acid and kept at 4 °C for 1 h. The plates were washed five times with cold distilled water and left to dry. 50 µl SRB solutions (0.4% w/v in 1% acetic acid) was added into each well, and the cells were allowed to stain for 10 min. The plates were washed three times with 1% acetic acid and were dried. Bound stain was dissolved with unbuffered 10 mM/L Tris base (tris-hydroxymethyl-aminomethane). Finally, the absorbance values were measured at 490 nm using a microplate reader (model 550, Bio-Rad, USA). Untreated cells were used as negative control. All SRB experiments were performed in triplicate. The cell proliferation inhibitory rate (IR) was calculated by the following formula: IR (%)=(1 - mean of tested SRB viability/mean of control SRB viability)×100%. The effect between TRAIL and cisplatin was analyzed according to previous method (29). This method provided a *q* value which was calculated using the following equation:* q *= E_A+B_/[(E_A_ +E_B_)-(E_A_×E_B_)], where E_A+B_, E_A_ and E_B_ represented respectively the IR of combination treatment, A only and B only. Then* q* ≤ 0.85, 0.85 ≤ *q* < 1.15, and *q *≥ 1.15 represented respectively antagonistic, additive and synergistic effects.

***Assessment of apoptosis***

Apoptosis morphology was observed by fluorescence microscope after Hoechst 33342 staining. Briefly, exponentially growing tumor cells were seeded into a 24-well plate (5×10^3^/well) for 24 h. Then cells were incubated with TRAIL alone, subtoxic-dose cisplatin alone or combination treatment in 5% CO_2_ at 37°C for 48 h. The supernatant was discarded and then cells were exposed to Hoechst 33342 (10 µg/ml) at 37°C in the dark for 20 min. Finally all specimens were observed under fluorescence microscope (Olympus AX80, Olympus). Apoptosis cells were identified as cells with condensed and fragmented nuclei.

The apoptosis rate was measured by flow cytometry after Annexin V-FITC and PI staining. Briefly, NCI-H460 or A549 cells from the above were collected, washed twice with cold phosphate-buffered solution (PBS), and permeabilized with 70% ethanol in PBS for 30 min. Then cells were resuspended in Annexin V binding buffer at a concentration of 5×10^5^/ml. FITC-conjugated Annexin V (1 μg/ml) and PI (50 μg/ml) were added to the cells, and incubated for 15 min in the dark. Quantitative analysis of apoptotic level was performed using an Elite-ESP flow cytometer (Beckman-Coulter, USA). A minimum of 10000 cells were analyzed in each sample, and all experiments were performed in triplicate.

***Reverse transcriptase polymerase chain reaction (RT-PCR) analysis***

The NCI-H460 or A549 cells were plated on a 6-well cluster dish (7.5×10^5^/well) for 24 hr. Medium containing TRAIL alone, subtoxic-dose cisplatin alone or combination treatment was added into the cells. After 48 hr incubation, total RNAs were extracted using Trizol reagent kit following the supplier instructions. Then RNAs were quantified spectrophotometrically. RT-PCR was run using One-step RT-PCR kit, and β-actin was used as internal control. The PCR primers were designed by Primer 5.0 software and the sequences were as follows: the upstream primers of DR4, DR5, DcR1, DcR2 and β-actin were 5’-CTGAGCAACGCAGACTCG CTGTCCAC-3’,5’-GCCTCATGGACAATGAGATAAAGGTGGCT-3’, 5’-GAAGAATTTGGTGCCAATGCCACTG-3’, 5’-CTTTTCCGGCGGCGTTCATGTCCTTC-3’ and 5'-AGGCAT CCTCACCCTGAAGTA-3', respectively. The downstream primers were 5’-TCCAAGGACACGGCAGAGCCTGTGCCAT-3’, 5’-CCAAATCTCAAAG TACGCACAAACGG-3’, 5’-CTCTTGGACTTGGCTGGGAGATGTG-3’, 5’-GTTTCTTCCAGGCTGCTTCCCTTTGTAG-3’, and 5’-AGCACAGCCTGGATAGCA A-3', respectively. The PCR products were a 506 bp for DR4 fragment, a 502 bp for DR5 fragment, a 612 bp for DcR1 fragment, a 453 bp for DcR2, and a 250 bp for β-actin fragment, respectively. PCR cycles included a denaturation step of 94°C for 25 sec, followed by an optimized annealing temperature of 56°C for 25 sec for DR4 and DR5 and 50°C for 25 s for DcR1 and DcR2, and a final elongation step at 72°C for 25 sec. The amplification cycles of DR4, DR5, DcR1 and DcR2 were repeated 25, 22, 30 and 30 times, respectively. PCR products were separated by electrophoresis using 1% agarose gel and visualized by ethidium bromide staining. The electrophoresis photo was transformed into computer, and DR4, DR5, DcR1, DcR2, and β-actin band integrated optical density values were analyzed using the Bio-Rad image system. Semiquantitative analysis of DR4, DR5, DcR1 and DcR2 mRNA was performed by comparison with β-actin.

***Western blotting***

The cells were harvested and protein content was measured using bicinchoninic acid (BCA) method. Cell lysate protein was subjected to 4%-12.6% gradient sodium dodecyl sulfate-polyacrylamide gel electrophoresis (SDS-PAGE) using a Tris-glycine system and gels were electroblotted onto PVDF membranes for 35 min. The membranes were incubated with 5% nonfat dry milk in PBS for 1 hr, and then incubated with the appropriate primary antibody concentration (1 : 400 dilution for Caspase-8, Caspase-9, Bcl-2 and Bax, 1:1000 for Dcr1 and DR4, 1:500 for DcR2 and DR5, 1 : 2000 for β-actin) at 37°C in 5% nonfat dry milk for 1.5 hr. Membranes were subsequently rinsed in PBS, and incubated with secondary antibody (goat antimouse IgG-AP or goat antirabbit IgG-AP, 1: 1000 dilution) at 37 °C for 1 h. The bound antibodies were visualized with BCIP/NBT. Signal density was obtained by scanning blots on a Bio-Rad imaging system. Normalized density was obtained by dividing the rough density values of a sample band over loading control band (β-actin). 

***Statistical analysis***

Data were compiled as the mean ± standard deviation (SD). Statistical comparisons were analyzed by one-way analyses of variance (ANOVA). All data were dealt with SPSS 14.0 statistical software. *P-*value below 0.05 was considered statistically significant.

## Results

***TRAIL inhibited proliferation in*** ***NCI-H4*** ***60 and A549*** ***cells***

**Figure 1:**
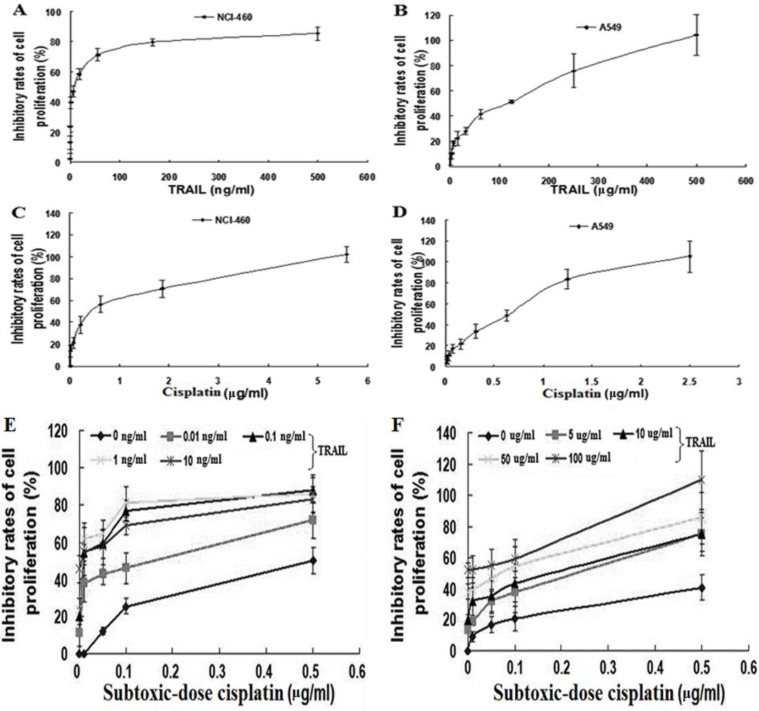
Growth inhibitory curve of TRAIL alone, cisplatin alone and TRAIL combined with subtoxic-dose cisplatin against NSCLC cells for 48 h: A, TRAIL alone for NCI-H460; B, TRAIL alone for A549; C, cisplatin alone for NCI-H460; D, cisplatin alone for A549; E, combination treatment for NCI-H460; F, combination treatment for A549. Data represented mean ± SD (n = 3)

To determine the effects of TRAIL on proliferation, NCI-H460 and A549 cells were treated respectively with 0.08~500 ng/ml and 2~500 μg/ml of TRAIL, and the proliferation IR was measured after 48 hr. As illustrated in Figure 1A and B, the growth inhibitory curves showed that 10.70 ng/ml of TRAIL resulted in 50% of IR in NCI-H460, indicating that NCI-H460 was sensitive to TRAIL. Otherwise, TRAIL caused also dose-dependent proliferation inhibition in A549, but 50% of IR was gained by 120.50 μg/ml of TRAIL. This result showed that only high concentration of TRAIL could result in improvement of IR, suggesting that A549 was resistant to TRAIL. These results revealed that NCI-H460 was sensitive to TRAIL; whereas, A549 was resistant. Further improved sensitivity of NSCLC cells to TRAIL-mediated growth inhibition was necessary, especially for A549.

***Cisplatin inhibited proliferation in*** ***NCI-H4*** ***60 and A549*** ***cells***

We next investigated the effects of cisplatin on proliferation. As described above, NCI-H460 and A549 cells were exposed to serial concentrations of cisplatin (0.01~5.6 μg/ml and 0.01~2.5 μg/ml, respectively) for 48 hr. The growth inhibitory curves revealed that cisplatin caused dose-dependent proliferation inhibition with the IC50 of 0.41 μg/ml and 0.62 μg/ml in NCI-H460 and A549, respectively (Figure 1C and D), suggesting that they were sensitive to cisplatin.

***S*** ***ubtoxic-dose cisplatin*** ***i*** ***ncreased TRAIL-induced p*** ***roliferation*** ***inhibition in*** ***NCI-H460 and A549*** ***cells***

To determine the effects of subtoxic-dose cisplatin plus TRAIL on proliferation, NCI-H460 and A549 cells were exposed to TRAIL alone, subtoxic-dose cisplatin alone or combination treatment for 48 hr, respectively. The concentrations of subtoxic-dose cisplatin were set as 0.01, 0.05, 0.1 and 0.5 μg/ml according to IC50 values obtained from above studies. Meanwhile 0.01, 0.1, 1, 10 ng/ml and 5, 10, 50, 100 μg/ml of TRAIL were used in NCI-H460 and A549, respectively. The growth inhibitory curves showed that combination treatment induced more significant increment of IR in both NCI-H460 and A549 cells compared to individual treatment with TRAIL or subtoxic-dose cisplatin (Figure 1E and F), suggesting that combination treatment had a synergistic effect. Almost all the *q *values were over 1.15 in NCI-H460 (Table 1), and seven *q* values were over 1.15, and other nine *q *values were between 0.85 and 1.15 in A549 (Table 2). In A549 the synergistic effect in combination treatment group occurred until subtoxic-dose cisplatin concentration reached 0.5 μg/ml. These results showed that subtoxic-dose cisplatin could apparently enhance TRAIL-induced proliferation inhibition in both TRAIL sensitive and nonsensitive NSCLC cells.

**Table 1:** Inhibitory rate of cell growth of subtoxic-dose cisplatin combmined with TRAIL against NCI-H460 cells. Data represent Mean ± SD (n = 3)

TRAIL		IR (%)of subtoxic-dose cisplatin (µg/ml) combined with TRAIL							
(ng/ml)	IR (%)	0.01	*q*	0.05	*q*	0.1	*q*	0.5	*q*
0	-	0.1±0.8	-	12.2±1.9	-	25.3±4.1	-	50.1±7.4	-
0.01	11.3±4.3	37.9±10.3	3.36^ a^	42.6±5.7	1.93^ a^	45.9±8.7	1.36^ a^	72.0±1.7	1.29^ a^
0.1	19.8±7.5	54.5±4.4	2.77^a^	59.0±7.4	2.00^ a^	77.0±5.4	1.92^ a^	88.0±7.0	1.47^ a^
1	23.7±5.8	61.9±8.5	2.63^ a^	64.4±1.3	1.96^ a^	80.9±4.8	1.88^ a^	86.0±10.4	1.39^ a^
10	45.6±7.5	54.9±13.0	1.21^ a^	57.7±9.6	1.10^b^	68.8±4.8	1.16^ a^	84.2±6.9	1.16^ a^

IR: inhibitive rate of cell growth. Antagonistic, additive, or synergistic effects were indicated when *q *< 0.85, 0.85 ≤ *q *≤ 1.15 or *q *> 1.15, respectively: a is representative of synergistic effect; b is representative of additive effect

***Subtoxic-dose cisplatin enhanced TRAIL-mediated apoptosis in NCI-H460 and A549 cells***

**Figure 2:**
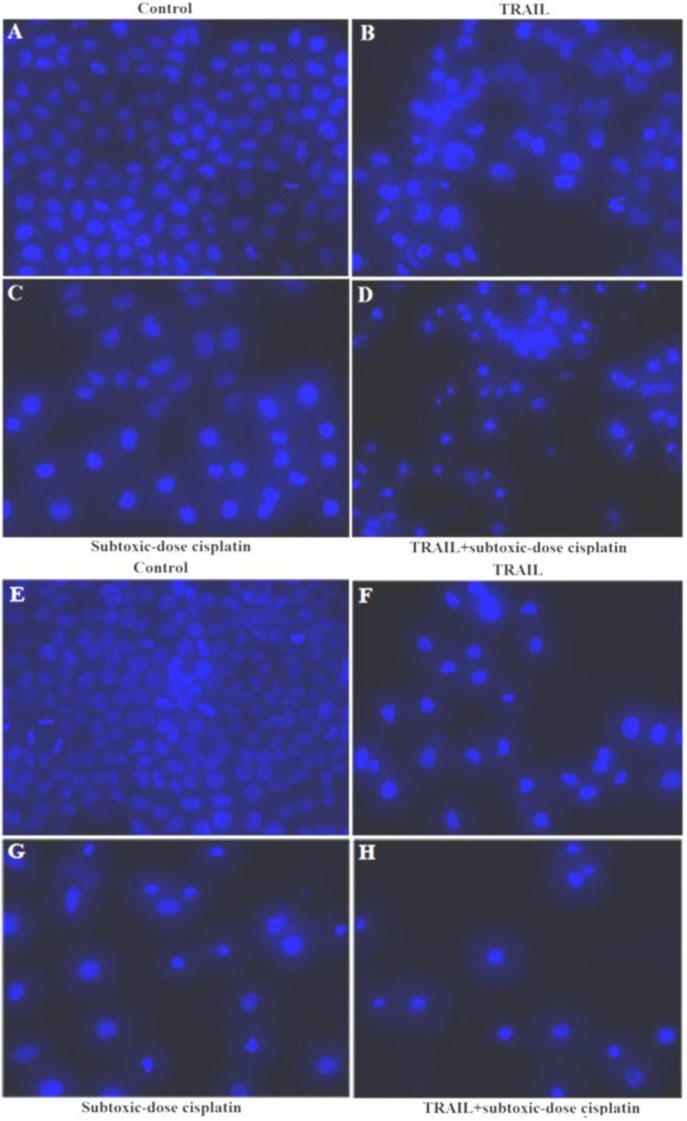
Apoptosis morphology in NSCLC cells treated with combination treatment by Hoechst staining ( 200): A-D, NCI-H460; E-H, A549; A, control; B, 1 ng/ml TRAIL; C, 0.1 μg/ml subtoxic-dose cisplatin; D, combination treatment; E, control; F, 5 μg/ml TRAIL; G, 0.5 μg/ml subtoxic-dose cisplatin; H, combination treatment

**Figure 3:**
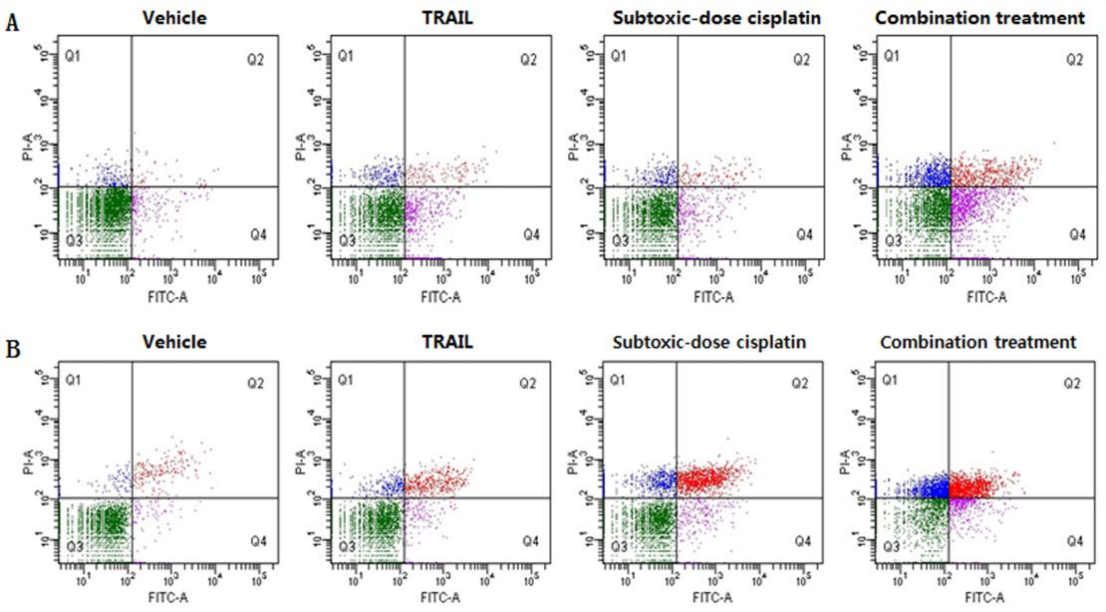
Subtoxic-dose cisplatin promoted TRAIL-mediated apoptosis of NSCLC cells *in vitro* and apoptosis was measured by FITC Annexin V and PI staining: A represented NCI-H460 cells; B represented A549 cells.

To further examine the effects of subtoxic-dose cisplatin on TRAIL-mediated apoptosis, NCI-H460 and A549 cells were also treated with TRAIL alone, subtoxic-dose cisplatin alone or combination treatment for 48 hr, respectively. The concentrations of subtoxic-dose cisplatin were set as 0.1 µg/ml and 0.5 µg/ml, and the concentrations of TRAIL were set as 1 ng/ml and 5 µg/ml in NCI-H460 and A549, respectively. The typical apoptotic morphology was observed in NCI-H460 (Figure 2A-D) and A549 (Figure 2E-H) cells and characterized by cell shrinkage, condensation and fragmentation of the nuclei as well as the apoptotic bodies. Furthermore, the result of FITC Annexin V and PI staining indicated that apoptosis rates in combination treatment group in NCI-H460 and A549 (26.9±3.5% and 41.1±10.6%, respectively) were significantly higher than those in subtoxic-dose cisplatin alone group (6.2±0.9%, *P*=0.001 and 22.5±1.0%, *P*=0.039, respectively) or TRAIL alone group (10.4±5.3%,* P*=0.011 and 8.2±1.4%,* P*=0.006, respectively) (Figure 3). Taken together, these results suggested that subtoxic-dose cisplatin could significantly enhance TRAIL-mediated apoptosis in NCI-H460 and A549 cells.

***Subtoxic-dose cisplatin combined with TRAIL affected TRAIL receptors levels in NCI-H460 and A549 cells***

**Figure 4:**
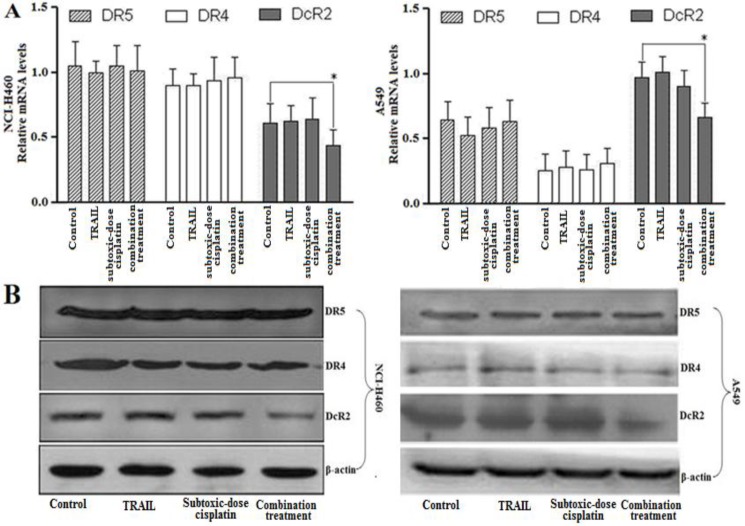
Subtoxic-dose cisplatin enhanced TRAIL-mediated apoptosis in NSCLC cells by down-regulating the DcR2 expression: A and B respectively represented the mRNA expression and protein level of DR4, DR5 and DcR2 for control, TRAIL alone (1 ng/ml for NCI-H460; 5 μg/ml for A549), subtoxic-dose cisplatin alone (0.1 μg/ml for NCI-H460; 0.5 μg/ml for A549) and combination treatment. Data represented mean ± SD (n = 3) (^*^, *P* < 0.05)

To determine the underlying mechanisms of subtoxic-dose cisplatin-stimulated TRAIL-mediated apoptosis in NCI-H460 and A549 cells, the mRNA and protein expression levels of TRAIL receptors were examined. As shown in Figure 4A, the mRNA of DcR2 in combination treatment group was significantly down-regulated compared to control group (n=3, *P*=0.023, NCI-H460 and *P*=0.015, A549). However, the mRNA of DR4 and DR5 was no significant difference between control and combination treatment groups in both NSCLC cells (n=3, *P*>0.05 in both NCI-H460 and A549). The mRNA of DcR1 in all groups could not be detected in both NSCLC cells. These results were further confirmed at protein expression levels (Figure 4B). Together, the down-regulation of DcR2 might be involved in the biological process that subtoxic-dose cisplatin enhanced NCI-H460 and A549 cells to TRAIL-mediated apoptosis.

**Table 2:** Inhibitory rate of cell growth of subtoxic-dose cisplatin combined with TRAIL against A549 cells. Data represent Mean± SD (n = 3)

TRAIL		IR(%) of subtoxic-dose cisplatin (µg/ml) combined with TRAIL							
(µg/ml)	IR (%)	0.01	*q*	0.05	*q*	0.10	*q*	0.50	*q*
0	-	9.1±6.2	-	16.8±5.4	-	20.6±7.9	-	40.3±5.1	-
5	13.6±2.4	18.5±1.8	0.86^ b^	31.6±18.0	1.13^ b^	37.6±13.6	1.20^ a^	75.3±4.1	1.56^ a^
10	19.7±9.3	30.5±9.2	1.13^ b^	35.5±10.8	1.07^ b^	43.5±12.4	1.20^ a^	74.9±10.9	1.44^ a^
50	37.7±4.9	39.2±7.9	0.90^ b^	46.1±4.1	0.96^ b^	58.2±2.7	1.15^ a^	85.4±6.7	1.28^ a^
100	51.8±5.0	52.3±8.8	0.93^ b^	54.8±10.5	0.91^ b^	59.2±6.4	0.96^ b^	109.9±8.8	1.61^ a^

IR: inhibitive rate of cell growth. Antagonistic, additive, or synergistic effects were indicated when *q *< 0.85, 0.85 ≤ *q *≤ 1.15 or *q *> 1.15, respectively: a is representative of synergistic effect; b is representative of additive effect

***Subtoxic-dose cisplatin combined with TRAIL affected intracellular*** ***apoptosis*** ***proteins*** ***levels*** ***in 549 cells***

**Figure 5:**
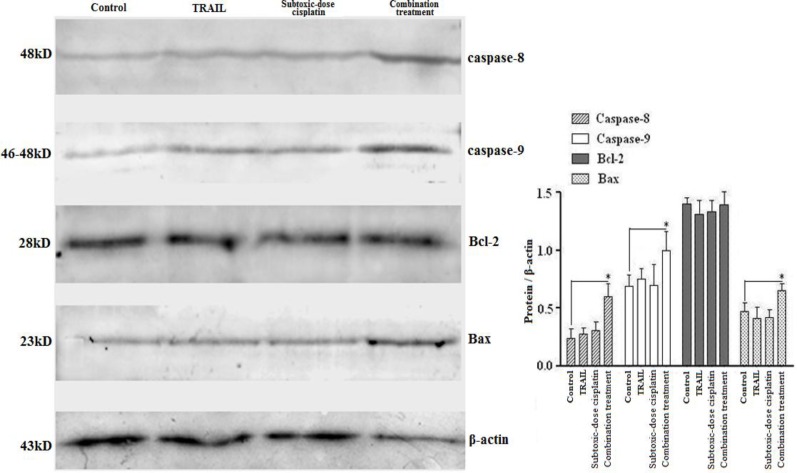
Subtoxic-dose cisplatin in combined with TRAIL affected intracellular apoptosis proteins levels of Caspase-8, Caspase-9, Bcl-2 and Bax in TRAIL-resistant A549. The concentration of subtoxic-dose cisplatin and TRAIL were respectively 0.5 μg/ml and 5 μg/ml. Data represented mean ± SD (n = 3) (^*^, *P* < 0.05).

Because A549 cells were more resistant to TRAIL compared to NCI-H460 cells, only A549 cells were selected in the following study. We then examined the protein expression levels of Caspase-8, Caspase-9, Bcl-2 and Bax which had been shown to be involved in apoptosis using Western blotting. As shown in Figure 5, combination treatment up-regulated significantly the expression of Caspase-8 (*P*=0.018), Caspase-9 (*P*=0.021) and Bax (*P*=0.036) compared to control, whereas it had no effect on the expression of Bcl-2 (*P*> 0.05). Furthermore, either TRAIL or subtoxic-dose cisplatin alone had no effect on these protein expression levels significantly. These results demonstrated that subtoxic-dose cisplatin might enhance TRAIL-mediated apoptosis through the up-regulation of Caspase-8, Caspase-9 and Bax expression.

## Discussion

Chemotherapy often causes unpleasant side effects including bone marrow inhibition, nausea and vomiting (30, 31). It has been reported that failure to control side effects can lead to 25%-50% of patients delaying or refusing possible life-saving therapy (31). Thus, novel treatment strategies are urgently needed to improve the clinical management including NSCLC treatment. During the first-line therapy for advanced NSCLC, platinum-based regimens have been the standard of care (32). Cisplatin, as the first generation platinum, has long been applied in clinical for its high activity in advanced NSCLC during three decades. However, routine dose of cisplatin often develops considerable toxicities after long-time treatment. Lower dose of cisplatin may avoid the toxicities, whereas the clinical effect may be also attenuated. Therefore, a strategy including low-dose cisplatin combined with other anticancer agents may bring new hope.

TRAIL exhibits potent antitumor activity with low side effects for normal cells (4, 5). However, some tumor cells show resistance to TRAIL. In this study, although NCI-H460 was found to be sensitive to TRAIL, IR was only slightly changed after the concentration of TRAIL was added to 100 ng/ml, which might be due to this issue that overabundant TRAIL failed to induce more generation of TRAIL receptors to further activate downstream pathway for killing cells. Meanwhile, A549 was found to be resistant to TRAIL. Treatment of A549 with 2 μg/ml TRAIL induced limited cytotoxicity (IR was approximately 10%), which was consistent with a previous report (33). Now we presumed that subtoxic-dose cisplatin could enhance NSCLC cells to TRAIL-mediated apoptosis to overcome TRAIL-resistance. Our results confirmed that subtoxic-dose cisplatin (0.5 µg/ml) could significantly enhance A549 and NCI-H460 cells to TRAIL-mediated apoptosis. The findings suggested that the combination treatment pattern of subtoxic-dose cisplatin and TRAIL might be potent for both TRAIL-resistant and TRAIL-sensitive NSCLC cells.

TRAIL-resistance might be attributable to down-expression of DR4 and DR5 or overexpression of DcR1 and DcR2 in tumor cells. Our results showed that NCI-H460 had high expressions of DR4 and DR5 and low expression of DcR2 on its surface (Figure 4); whereas, A549 exhibited opposite expression pattern of the three genes. This different expression pattern of TRAIL receptors on cell surface in NSCLC cells might be involved in their different responses to TRAIL. These data were consistent with previous report about breast cancer cells (34). 

DcR1 and DcR2 are unable to directly induce apoptosis due to lack of intact death domain in the intracellular, but they could compete with DR4 and DR5 for binding TRAIL (35). Thus, overexpression of DcR1 and/or DcR2 could block TRAIL-mediated apoptosis (7). Previous studies had reported that low-dose anticancer agents could sensitize cancer cells to TRAIL-mediated apoptosis, but they did not significantly alter DR4 and DR5 expression (36). Meanwhile silencing DcR2 by siRNA could lead to a significant increment in TRAIL-mediated apoptosis (12). To investigate the underlying mechanism of synergistic effect of subtoxic-dose cisplatin with TRAIL in NSCLC cells, we examined the expression of TRAIL receptors. Our results revealed that combination treatment could down-regulate DcR2 expression, but did not affect DR4 and DR5 expression. The reason was presumed that the down-regulation of DcR2 weakened binding activity of NSCLC cells with TRAIL through DcR2; whereas, strengthened their binding activity with TRAIL through DR4 and/or DR5. Then we further examined the protein expression levels of Caspase-8, Caspase-9, Bcl-2 and Bax. The results showed that combination treatment significantly up-regulated the expression of Caspase-8, Caspase-9 and Bax. It seems that apoptosis was not mediated by transcriptional induction of DR4 and DR5; whereas, caspase activation from other pathways might be involved in apoptosis mechanism. In conclusion, our findings motivated further studies to evaluate the combinatory therapeutic strategy of subtoxic-dose cisplatin and TRAIL against NSCLC in the animal models.

## Conclusion

The data obtained in the present study indicated that subtoxic-dose cisplatin could enhance both TRAIL- sensitive and TRAIL- resistant NSCLC cells to TRAIL-mediated cell proliferation inhibition and apoptosis. The down-regulation of DcR2 and up-regulation of Caspase-8, Caspase-9 and Bax might be involved in its underlying mechanisms.
